# Levodopa-responsive dystonia caused by biallelic *PRKN* exon inversion invisible to exome sequencing

**DOI:** 10.1093/braincomms/fcab197

**Published:** 2021-09-06

**Authors:** Hagar Mor-Shaked, Emuna Paz-Ebstein, Adily Basal, Simona Ben-Haim, Hanna Grobe, Sami Heymann, Zvi Israel, Montaser Namnah, Anat Nitzan, Chaggai Rosenbluh, Ann Saada, Tomer Tzur, Shira Yanovsky-Dagan, Ronen Zaidel-Bar, Tamar Harel, David Arkadir

**Affiliations:** 1 Department of Genetics, Hadassah Medical Organization, Jerusalem 91120, Israel; 2 Faculty of Medicine, Hebrew University of Jerusalem, Jerusalem 91120, Israel; 3 Department of Nuclear Medicine, Hadassah Medical Organization, Jerusalem 91120, Israel; 4 Institute of Nuclear Medicine, University College London and UCL Hospitals, NHS Trust, London NW1 2BU, UK; 5 Department of Cell and Developmental Biology, Faculty of Medicine, Tel Aviv University, Tel Aviv-Yafo 69978, Israel; 6 Department of Neurosurgery, Hadassah Medical Organization, Jerusalem 91120, Israel; 7 Department of Neurology, Hadassah Medical Organization, Jerusalem 91120, Israel; 8 Department of Plastic Surgery, Hadassah Medical Organization, Jerusalem 91120, Israel

**Keywords:** *PRKN*, *PARK2*, dystonia, Parkinson's disease, structural variants

## Abstract

Biallelic pathogenic variants in *PRKN* (*PARK2*), encoding the E3 ubiquitin ligase parkin, lead to early-onset Parkinson's disease. Structural variants, including duplications or deletions, are common in *PRKN* due to their location within the fragile site FRA6E. These variants are readily detectable by copy number variation analysis. We studied four siblings with levodopa-responsive dystonia by exome sequencing followed by genome sequencing. Affected individuals developed juvenile levodopa-responsive dystonia with subsequent appearance of parkinsonism and motor fluctuations that improved by subthalamic stimulation. Exome sequencing and copy number variation analysis were not diagnostic, yet revealed a shared homozygous block including *PRKN*. Genome sequencing revealed an inversion within *PRKN,* with intronic breakpoints flanking exon 5. Breakpoint junction analysis implicated non-homologous end joining and possibly replicative mechanisms as the repair pathways involved. Analysis of cDNA indicated skipping of exon 5 (84 bp) that was replaced by 93 bp of retained intronic sequence, preserving the reading frame yet altering a significant number of residues. Balanced copy number inversions in *PRKN* are associated with a severe phenotype. Such structural variants, undetected by exome analysis and by copy number variation analysis, should be considered in the relevant clinical setting. These findings raise the possibility that *PRKN* structural variants are more common than currently estimated.

## Teaser

Mor-Shaked et al. report a homozygous copy number neutral inversion in *PRKN,* encoding parkin, in a family with juvenile levodopa-responsive dystonia and parkinsonism. The authors demonstrate that the inversion leads to exon skipping and intronic inclusion, and highlight the importance of whole-genome sequencing in exome-negative cases.

## Graphical Abstract

**Graphical Abstract:**
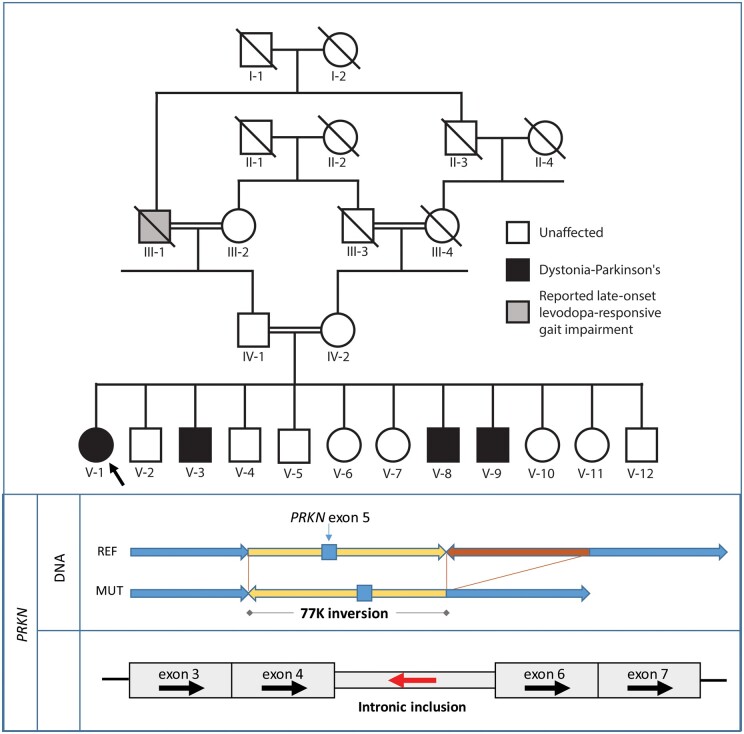

## Introduction

Biallelic pathogenic variants in the *PRKN* (*PARK2*, [MIM 600116]) gene, encoding the E3 ubiquitin ligase parkin, are often detected in juvenile or young-onset Parkinson's disease.^1^^,^^2^ Focal dystonia, mainly of the lower limb, is a frequent presenting symptom of *PRKN*-associated Parkinson's disease.^2^^,^^3^ In severe cases, this focal dystonia rapidly progresses to generalized levodopa-responsive dystonia.^3^^,^^4^ In such cases, the diagnosis of Parkinson's disease becomes more obvious once motor fluctuations are observed and may be further supported by nuclear imaging demonstrating dopaminergic striatal deficit.

In up to 60% of the cases, *PRKN*-associated Parkinson's disease is caused by copy number variants (CNVs),^5^ either large deletions or duplications of entire exons.^2^^,^^6^ These variations, detected by read depth CNV analysis or by multiplex ligation-dependent probe, represent multiple independent rearrangement events.^5^^,^^7^^,^^8^

We describe here a copy-number neutral intragenic inversion in *PRKN*. This structural variant, undetected by exome sequencing (ES) and CNV analysis, is associated with a severe phenotype of juvenile Parkinson's disease first presenting as generalized levodopa-responsive dystonia.

## Materials and methods

### Study participants

The study was approved by the local institutional review board and all participants signed informed consent forms before beginning the study (0393–17-HMO).

### Electrophysiological recording

Deep brain stimulation surgery was performed under general anaesthesia (induction with propofol, followed by ketamine). Electrophysiological data were acquired using the Neuro Omega system (Alpha Omega, Nazareth, Israel).

### Exome sequencing

DNA samples, obtained from peripheral blood, were prepared and processed for ES as previously described.^9^ Briefly, exonic sequences were enriched in the DNA sample using SureSelect Human All Exon 50 Mb Kit V5 (Agilent Technologies, Santa Clara, CA, USA) and sequenced using a HiSeq2500 or NovaSeq6000 sequencing system (Illumina, San Diego, CA, USA) as 100- or 150-bp paired-end runs. Reads were aligned to the reference human genome assembly hg19 (GRCh37) using the Burrows-Wheeler Alignment (bwa) Tool v 0.7.10.^10^ Variants were called using the GATK-lite pipeline v4^11^ and were annotated using Ensembl Variant Effect Predictor.^12^ Following alignment to the reference genome and variant calling, variants were filtered out if the total read depth was less than 8×, they were off-target (>8 bp from splice junction), synonymous (unless <3 bp from splice junction), or had a minor allele frequency >0.01 in the gnomAD database or in our in-house exome database comprising 7000 exomes. Exomes of the affected siblings yielded 93–100 million reads, with a mean coverage of 110–120×, and >97% over 20×. Exomes of the healthy siblings were sequenced at low coverage (20–30×).

### CNV analysis

CNV analysis was performed using an in-house python script, based on read depth data extracted from the GATK tool CollectHsMetrics. The ratio between the normalized read depth of the exome of interest and those of a reference group (>70 controls) was used to indicate suspected deletion or duplication events. The script is available upon request.

### Whole-genome sequencing

Genomic DNA was extracted from peripheral blood and NGS libraries were prepared with an Illumina PCR-free TruSeq DNA Library Prep Kit. Sequences were generated on an Illumina NovaSeq 6000 sequencing platform as 150 bp paired-end reads, to a final depth of 30X coverage. The FASTQs were uploaded into the Geneyx (previously TGex) Analysis platform.^13^ Alignment and variant calling of single nucleotide variations, structural variants, CNVs and repeats were called using Illumina DRAGEN Bio-IT. The resulting VCF files were comprehensively annotated on the Geneyx Analysis annotation engine, and presented for analysis, filtering and interpretation. Variant prioritization was performed using VarElect.^14^

### Runs of homozygosity analysis

Detection of runs of homozygosity in individual exomes was performed using ‘DetectRuns’ ^15^ with the following parameters: window size 50 SNPs, minimal length of block 5 Mb, minimal number of homozygous SNPs in a block 80, the maximum number of heterozygous SNPs in a window 3, the maximum gap between consecutive SNPs 1.5 Mb, SNPs density 1/90 and minimum block size of 1.5 Mb. Homozygous mapping of the runs of homozygosity segments was visualized using the karyoploteR package.^16^

### Tissue cultures

Fibroblasts from skin biopsies were cultured in DMEM (Biological Industries Beit Haemek, Israel) with 15% foetal bovine serum, penicillin–streptomycin and l-glutamine at 37°C in 5% CO_2_.

### Breakpoint junction analysis

The distal breakpoint junction was amplified by the following primers: PRKN_jct2_F1: 5′-TGG AAC AAA CAC CGC TAT CA-3′ and PRKN_jct2_R1: 5′-TTT CTC CTG GTT GTG GTT CC-3′.

### RNA/cDNA analysis

RNA was isolated from patient fibroblasts by TRIzol reagent extraction and cDNA was prepared from 1 μg RNA using the qScript cDNA Synthesis Kit (Quantabio). The region encompassing exons 3–7 of *PRKN* was amplified by PCR reaction using Platinum SuperFi DNA Polymerase Master Mix (Invitrogen) with forward primer: PRKN_cDNA_F1: 5′-GCA GAG ACC GTG GAG AAA AG-3′ and reverse primer: PRKN _cDNA_R1: 5′-AAG GCA GGG AGT AGC CAA GT-3′. The resultant fragments were separated by agarose gel electrophoresis and their sequences were determined by Sanger sequencing.

### Data availability

The *PRKN* variant was submitted to ClinVar (accession number VCV001027679.1).

## RESULTS

### Clinical description

**Figure 1:**
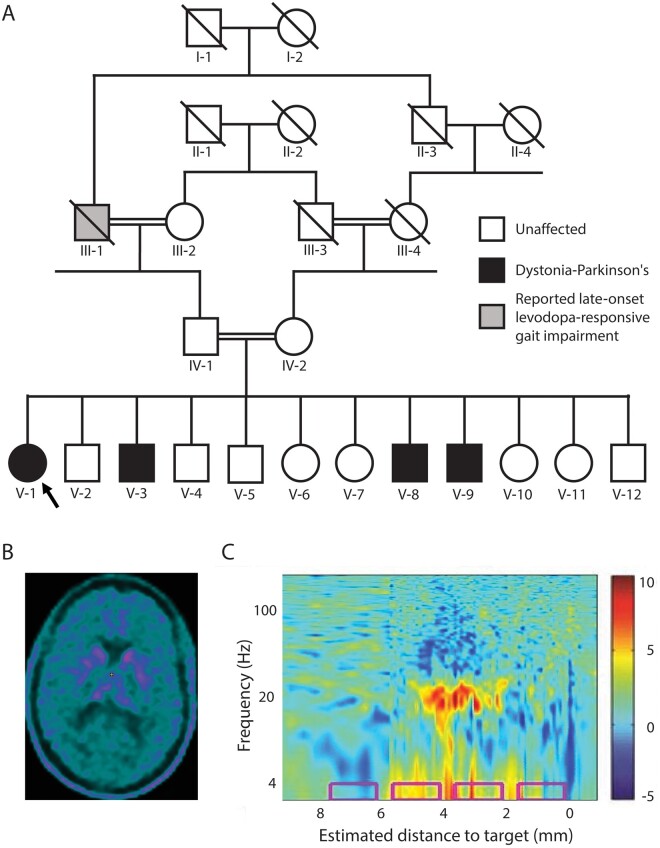
Clinical characteristics (**A**) Family tree of the studied family. Proband is indicated by an arrow. (**B**) Brain ^18^F-l-3,4-dihydroxyphenylalanine (^18^F-DOPA) PET/CT study (patient V-1) demonstrating severely impaired pre-synaptic striatal dopaminergic integrity. (**C**) Power spectral density plot demonstrates increased β oscillatory activity (around 20 Hz) in the right subthalamic trajectory of patient V-3, as a function of distance along the surgical trajectory. Estimated distance to target was preoperatively defined based on MRI. The colour scale represents log10 of power spectral density/average power spectral density. The implanted final contact depths of the permanent electrode are depicted by the pink rectangles.

The studied family is a consanguineous family of Yemenite-Jewish origin (Fig. 1A). Four of 12 siblings developed young onset levodopa-responsive dystonia (median age 11 years, range 9–14, Supplementary Table 1). The first reported symptom was dystonic foot inversion during walking in three individuals and upper limb action dystonia with a tremor in the fourth individual. Within few years, the dystonia gradually generalized to the other limbs (initially to the ipsilateral limb) and then to the trunk and neck in all affected individuals. Oculogyric dystonia or blepharospasm were not observed. Diurnal variation of symptoms was not reported. All dystonic features were levodopa-responsive and improved dramatically shortly (20–40 min) after oral levodopa. Significant clinical response was reported with 125–150 mg of daily levodopa.

A few years after the onset of symptoms (reported age 13–19 years), levodopa-responsive parkinsonian features (asymmetric bradykinesia, resting tremor and severe rigidity and impaired gait) also appeared in all affected individuals. All four individuals started to experience motor fluctuations in their early twenties, which gradually progressed and included both early wearing-off and peak-dose dyskinesia involving all four limbs and trunk and indistinguishable from these of idiopathic Parkinson's disease. During their ‘off’ periods general severe dystonia and rigidity re-emerged with prominent painful opisthotonos in the two elderly affected individuals. Asymmetrical pyramidal signs (brisk lower limb reflexes and left ankle clonus) were observed in a single patient (V-8).

Due to these severe motor fluctuations, the three most affected individuals (V-1, V-3 and V-8) underwent bilateral subthalamic deep brain stimulation surgery (at the age 35, 33 and 22 years, respectively). Brain stimulation led to a dramatic improvement in both the parkinsonian signs and the dystonia (including the opisthotonos).

Two affected individuals (V-1, V-3) had ^18^F-l-3,4-dihydroxyphenylalanine brain PET/CT studies in order to evaluate the integrity of their striatal dopaminergic innervation. These studies demonstrated severely impaired striatal presynaptic uptake of ^18^F-l-3,4-dihydroxyphenylalanine (Fig. 1B). Brain MRI studies of both individuals were unremarkable.

The extracellular activity in the subthalamic nuclei was recorded during the surgery for electrode implantation. In patient V-1, operated in partial ‘on’ state, oscillatory activity in the beta-range was not observed. In patients V-3 and V-8, operated in the ‘off’ state, prominent oscillatory activity in the beta-range, compatible with dopamine deficiency, was recorded (Fig. 1C).

### Genetic analysis

ES was performed in the four affected and in four healthy siblings. Homozygous variants of interest are listed in Supplementary Table 2. Two homozygous variants, the first in *TCP1* (T-Complex 1) and the second in *MAS1* (MAS1 Proto-Oncogene, G Protein-Coupled Receptor), both within the same homozygous block in chromosome 6, were found in a homozygous state in only one additional family member, a single sibling without symptoms but with right wrist lead-pipe rigidity on neurological examination (V-5). The possibility that one of these two variants was the cause of the disease, and that a very mild phenotype on V-5 was part of the clinical spectrum, led to extensive functional studies of *TCP1* which did not support pathogenicity (data not shown). This prompted us to pursue further genomic analysis.

**Figure 2:**
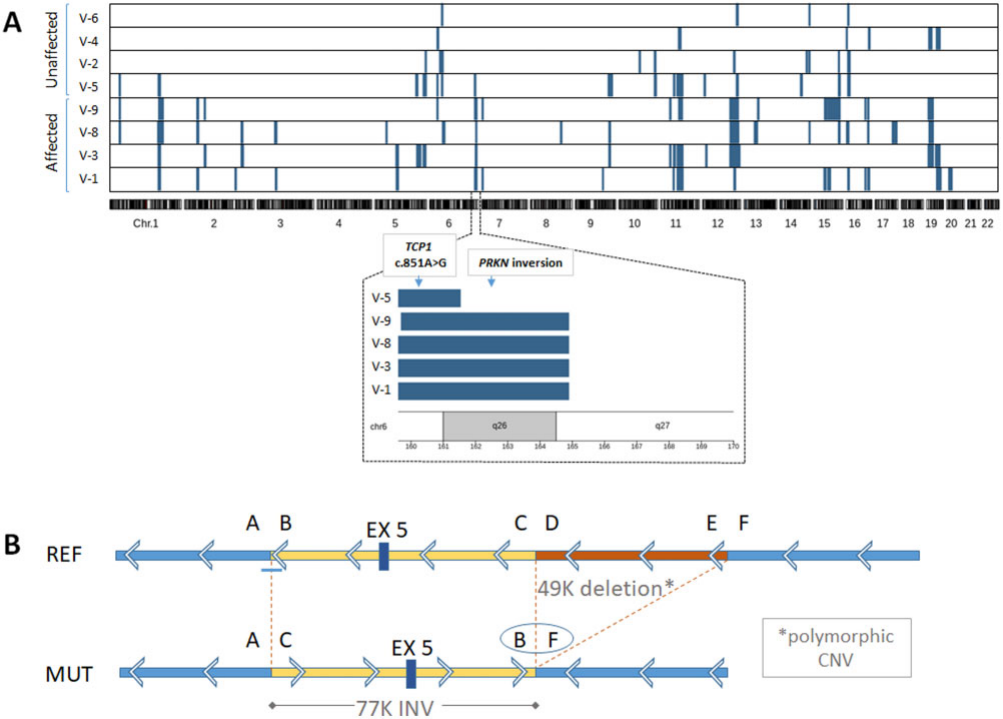
Runs of homozygosity analysis and characterization of the PRKN inversion at the DNA level. (**A**) The x-axis represents genomic position, ordered according to chromosome number. Each row in the figure represents a single individual. Homozygous blocks are marked in blue. One homozygous block (chr6:159655593–164891969; marked by a dashed line on chromosome 6) was shared by all four affected individuals and not by the unaffected ones, except the healthy sibling V-5. Zooming in this block (lower panel) shows that it was shorter in V-5 in comparison to the affected siblings, and did not include the *PRKN* gene. Homozygous mapping of runs of homozygosity segments were visualized using the karyoploteR package. (**B**) Schematic representation of the mutant sequence (lower panel) compared to the reference genome (upper panel). Arrows depict orientation of the *PRKN* gene, which is transcribed from the minus strand. The AC and BF coordinates represent the breakpoint junctions of the 77K inversion. The DE sement represents a 49K deletion which is common in the population. Genomic locations of the coordinates are as follows: A-chr6:162432171(+), B-chr6:162432262(−), C-chr6:162509558(−), D-chr6:162559511(+). CNV = copy number variation; INV = inversion.

Linkage analysis based on runs of homozygosity, performed on the ES data, revealed a single 10.5 kb homozygous block, containing the *PRKN* gene, shared by all clinically affected siblings (chr 6:159655593–164891969[hg19]). Individual V-5 (healthy) was homozygous for most of this block, including at the *TCP1* gene, yet underwent meiotic recombination within the *PRKN* gene (Fig. 2A). ES, however, did not detect potentially pathogenic single nucleotide variations nor CNVs^2^^,^^6^ in *PRKN*.

We therefore proceeded with whole-genome sequencing (WGS) on one of the affected siblings (V-1) and the healthy sibling (V-5). WGS analysis revealed a homozygous inversion of exon 5 of *PRKN* in the affected individual (Fig. 2B) followed by a common 49 kb deletion (demonstrated in 75 individuals, frequency 0.3, in the Database of Genomic Variants), which was found to be heterozygous in the healthy sibling. The proximal breakpoint (junction A–C in Fig. 2A), as defined by WGS split-read analysis (Supplementary Table 3), showed a 10-bp insertion (Supplementary Fig. 1A); this breakpoint could not be mapped by Sanger sequencing due to technical difficulties of a repetitive region. The 10-bp insertion was possibly templated by a sequence ∼150 downstream of breakpoint junction A, with an identity of 9 of the 10 inserted nucleotides (chr6:162432314–162432322), suggested replication-based mechanisms (such as fork stalling and template switching/microhomology-mediated break-induced replication, FoSTeS/MMBIR) as a possible repair mechanism at the proximal junction. The distal breakpoint (junction B–F in Fig. 2B and Supplementary Fig. 1B and C) was confirmed by Sanger sequencing and revealed a blunt end mutational signature, suggestive of non-homologous end-joining as the repair mechanism. Breakpoints were located within or in proximity to repetitive regions (Supplementary Table 4), yet the repeats were not homologous, ruling out non-allelic homologous recombination as a mechanism.^17^ The meiotic recombination event in Individual V-5, mapping proximal to the inversion, may be related to a possible meiotic recombination hot spot in this region.^18^ Moreover, heterozygosity for the inversion probably imposes a risk for an adult-onset Parkinson's disease and may explain his right wrist rigidity.

**Figure 3:**
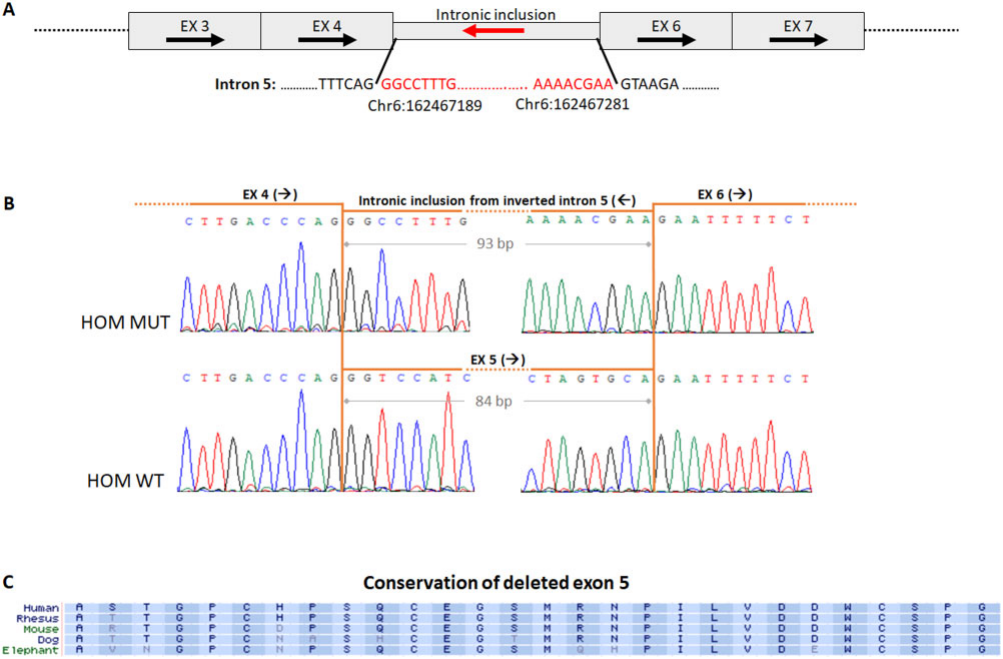
**Characterization of the PRKN inversion at the RNA level.** (**A**) Schematic representation of the mutant transcript, illustrating the intronic inclusion from inverted intron 5 (red arrow). Arrows depict native orientations. (**B**) Sanger sequencing of mutant (upper panel) and wild type (lower panel) RT-PCR products from patient V-1. In the mutant transcript, the deleted exon 5 (84 bp) is replaced by 93 bp from intron 5. (**C**) Amino acid conservation of exon 5 in various species. EX = exon; HOM = homozygous; MUT = mutant; WT = wild type.

In order to establish the effect of the *PRKN* inversion at the RNA level, we analysed the cDNA sequence derived from patient fibroblasts (individual V-1). RT-PCR revealed the existence of an amplicon with a molecular weight that approximated that of controls. Sanger sequencing demonstrated skipping of exon 5 (84 bp), and an in-frame intronic inclusion of 93 bp from within the sequence of the inverted intron 5 (Fig. 3A and B). The 93 bp sequence from within the inverted intron was flanked by consensus splice-sites (Supplementary Fig. 2), explaining why this region was erroneously recognized as an exon. Overall, the 28 relatively conserved amino acids of the native exon 5 (Fig. 3C) were replaced by 31 alternate residues (in frame), presumably leading to dysfunction of the encoded protein.

## DISCUSSION

Biallelic pathogenic variants in the *PRKN* gene are the most common cause of early-onset Parkinson's disease.^2^ We describe here a copy-neutral intragenic inversion in *PRKN*, undetected by ES and CNV analysis. This finding raises the possibility the variants in the PRKN gene account for a higher number of young-onset Parkinson's disease cases than previously estimated.

*PRKN* is located within FRA6E, one of the most unstable common fragile sites of the human genome.^19^ The main fragility core of FRA6E is localized to the region spanning exons 2 to 8 of *PRKN*, rendering this region a mutational hotspot.^20^ Ambroziak et al. described a duplication of exons 2 through 5 of *PRKN* in inverted orientation in one of the alleles of a compound heterozygous individual with early-onset Parkinson's disease (onset 33 years). While this variant would be detected by CNV analysis, in the case presented herein a smaller, non-duplicated inversion of a single exon required a different approach. Large inversions are known as the genetic cause of several medical conditions. For example, the most common cause underlying Haemophilia A (Factor VIII deficiency) is a recurrent inversion in the *F8* gene, mediated by inverted segmental duplications. Other examples include inversions leading to Hunter syndrome and to Emery-Dreifuss muscular dystrophy.^21^

The mutational signatures at the breakpoint junctions (i.e. a blunt end at one junction and a 10-bp insertion at the other junction) implicate double-stranded DNA breaks with non-homologous end joining and possibly replicative-based mechanisms such as FoSTeS/MMBIR, as the underlying mechanisms. Here, nearby repeats were not homologous (Supplementary Table 4) rendering them unlikely to have been drivers of the rearrangement. This is consistent with previous studies suggesting non-homologous end joining and FoSTeS/MMBIR as the major mechanisms responsible for the majority of the structural variants at this locus.^5^^,^^8^^,^^21^^,^^22^^,^^23^ It has also been proposed that retrotransposition events might trigger structural variation in *PRKN,* based on retrotransposons (Alu elements and long terminal repeats) identified within 1–2 kb of the breakpoints.^24^

The affected members of the family presented here have a relatively severe phenotype. While the median age of PRKN-PD onset is 31 years^25^ in the family described here it was 11 years (lower 2% of disease-free curve). In addition, while limb dystonia is not an uncommon presentation PRKN-PD^2^^,^^3^^,^^26^ generalized dystonia is much less frequent.^3^^,^^4^ Based on our results, and the results of others,^4^ it seems that a more severe dystonia is associated with a younger age of symptom onset and tends to occur in the first or second decades of life. Before Parkinsonian signs and motor fluctuations appear, it is not trivial to clinically distinguish these cases from non-degenerative dopamine-responsive dystonia (e.g. due to mutations in GTP cyclohydrolase I). Imaging of the striatal dopaminergic innervation, such as the ^18^F-l-3,4-dihydroxyphenylalanine scan that was used here, can facilitate diagnosis since innervation is largely preserved in non-degenerative dystonia.^27^ In addition, other metabolic aetiologies of young-onset dystonia, many of them also causing parkinsonism, should be considered in the differential diagnosis. These include Wilson disease and sepiapterin reductase deficiency,^28^^,^^29^ which are treatable diseases and therefore must not be misdiagnosed.

Commonly-used methods used to diagnose PRKN-PD include ES combined with either read-depth CNV analysis or with multiplex ligation-dependent probe. These methods are insufficient to detect intragenic inversions, such as the one presented here. Other ES limitations are the inability to reliably detect repeat-expansion diseases, mitochondrial disorders and copy-neutral recombinations. Moreover, the detection of a potential variant of unknown significance in a candidate gene may trigger futile functional studies before proceeding to WGS. This may be even more challenging if such variants are detected in genes that are physically linked to *PRKN*. Considering the high cost of WGS, it may not be practical to recommend this as an initial diagnostic test. We suggest performing WGS with either short or long reads in any case of undiagnosed young-onset dopamine-responsive dystonia or young-onset Parkinson's disease refractory to ES and CNV analysis, especially in cases where the *PRKN* gene is positioned in a homozygous block or where a single heterozygous variant was found and the second hit is sought.

## Supplementary material

Supplementary material is available at *Brain Communications* online.

## Competing interests

The authors declare that there is no conflict of interest. 

## Supplementary Material

fcab197_Supplementary_Data

## Abbreviations

CNV =: copy number variants
ES =: exome sequencing
PD =: Parkinson's disease
