# Supplementary material for: Levodopa-responsive dystonia caused by biallelic PRKN exon inversion invisible to exome sequencing
Source: Brain Commun. 2021 Sep 6;3(3):fcab197. doi: 10.1093/braincomms/fcab197 (PMC8421701; doi:10.1093/braincomms/fcab197)

**[SUPPLEMENTARY TABLES](https://oup.silverchair-cdn.com/oup/backfile/Content_public/Journal/brain/PAP/10.1093_brain_awaa445/1/awaa445_supplementary_data.zip?Expires=1614478480&Signature=zA5GDuv0wpkDnsUql8KWYenkpKj5ZZqMeKxBROasfWoQpJKz6hG3qkjHYl7yMEo7PztIL0MCfZiPdVU8iLCeGynS05oaIOGL0Oyu4CW~~3aQEcUhJqvA8ebrNvzIz95DqkzFCTG~bf7kXFb3dN7ntsZdLZKxbPWjTQP3KVNvGarmGH~s3TYQD1Wa0wwICQgEet2h5K6Vj548qdCfv8auKKydRDJzif0VXN7xACdGtQePmJuMHUglayO0RDkQhMjkonXv~QyTL6gSqXNPOhctG78aqLgpiWwzd05GYTx3grKJrGuz2Dg2-lr1MzO12KnswNi6fg33DXjxRNFg7ank~A__&Key-Pair-Id=APKAIE5G5CRDK6RD3PGA)**

**Supplementary Table 1: Additional clinical information**

| Individual | Dystonia - reported age of onset (years) | Parkinsonism - reported age of onset (years) | Motor fluctuations - reported age of onset (years) | Age at examination (years) | UPDRS III OFF | UPDRS III ON |
| --- | --- | --- | --- | --- | --- | --- |
| V-1 | 14 | 19 | 21 | 33 | NA | NA |
| V-3 | 9 | 16 (?) | 21 | 32 | 42 | 12 |
| V-8 | 13 | 15 (?) | 21 | 22 | 59 | 20 |
| V-9 | 9 | 13 (?) | 20 | 20 | NA | NA |

UPDRS III: Unified Parkinson's Disease Rating Scale part 3 (motor).

**Supplementary Table 2: Shared homozygous variants of unknown significance identified by exome sequencing**

| **No.** | **Chromosomal Position** | **dbSNP** | **RefSeq** | **Gene** | **HGVSc** | **HGVSp** | **GERP** | **CADD score** | **GnomAD Allele Frequency** |
| --- | --- | --- | --- | --- | --- | --- | --- | --- | --- |
| 1 | Chr6:160202089 | rs777637046 | [NM_030752.3](https://varsome.com/transcript/hg19/NM_030752.3) | *TCP1* | c.851A>G | p.Asn284Ser | 5.9 | 22.7 | 0.0012% |
| 2 | Chr6:160328898 | rs748801784 | [NM_002377.4](https://varsome.com/transcript/hg19/NM_002377.4) | *MAS1* | c.911A>G | p.Lys304Arg | 5.53 | 23.3 | 0.0012% |
| 3 | Chr16:66434688 | rs376438856 | [NM_001795.5](https://varsome.com/transcript/hg19/NM_001795.5) | *CDH5* | c.1606A>G | p.Ile536Val | 3.08 | 19.83 | 0.0008% |
| 4 | Chr19:34302285 | rs201658339 | [NM_001129995.2](https://varsome.com/transcript/hg19/NM_001129995.2) | *KCTD15* | c.521C>T | p.Thr174Met | 5.02 | 24.4 | 0.0065% |

**Supplementary Table 3: Split Reads**

| **Alignment 1** | **Alignment 2** | **Orientation** |
| --- | --- | --- |
| chr6:162432060-162432171 | chr6:162509529-162509558 | TAATTATAATATATATTTATAATATATATTATAATATATAATATATTTATAATATATATTATAATATATAATATATTTATAATATATAATATATTTATAATATATAATTATACAAAATATGTACAAAATTTTGACTAAAATTTTACTAAGCC |
| chr6:162432098-162432171 | chr6:162509515-162509558 | TATTTATAATATATATTATAATATATAATATATTTATAATATATATTATAATATATAATATATTTATAATATATAATATATTTATAATATATAATTATACAAAATATGTACAAAATTTTGACTAAAATTTTACTAAGCCAATTTAATTTTTTC |
| chr6:162432262-162432320 | chr6:162559511-162559603 | ATATTTTATAAATATATATTATAAATATATACTATATTATATATATTATAAATTATATAGTACAATGTCTGGAATCTAGTAAATCACTATGAATCGTGCAAAGAAACATAAAAATAGGAACCACAACCAGGAGAAAAATCAATCAATTAAAA |
| chr6:162432262-162432331 | chr6:162559511-162559592 | TACTATATTACATATTTTATAAATATATATTATAAATATATACTATATTATATATATTATAAATTATATAGTACAATGTCTGGAATCTAGTAAATCACTATGAATCGTGCAAAGAAACATAAAAATAGGAACCACAACCAGGAGAAAAATCA |

**Supplementary Table 4: Details of breakpoint junctions**

| **Breakpoint junction** | **Chromosomal Position(proximal)** | **Orientation** | **Repeat element (proximal)** | **Chromosomal Position (distal)** | **Orientation** | **Repeat element (distal)** | **Mutational signature** |
| --- | --- | --- | --- | --- | --- | --- | --- |
| A-C | Chr6:162432171 (hg19)  Chr6: 162011139 [hg38] | Plus | (TA)n, family Simple_ Repeat | Chr6:162509558 [hg19[  Chr6:162088526 [hg38] | minus | (in proximity to L1MB7) | insertion 10bp |
| B-F | Chr6:162432262 [hg19]  Chr6: 162011230 [hg38] | minus | (TA)n, family Simple_ Repeat | Chr6:162559511 [hg19]  Chr6:162138479 [hg38] | Plus | L1MEc, family L1 | blunt end |

**SUPPLEMENTARY FIGURES**

**Supplementary Figure 1: Characterization of the *PRKN* inversion at the DNA level.** (A) Breakpoint junction sequence of junction AC, indicating an insertion of 10bp at the breakpoint. Data is derived from split reads of WGS analysis. (B) Sanger sequencing of the distal breakpoint junction (BF) of the inversion. (C) Breakpoint junction sequence of junction BF, indicating the blunt end mutational signature.

**
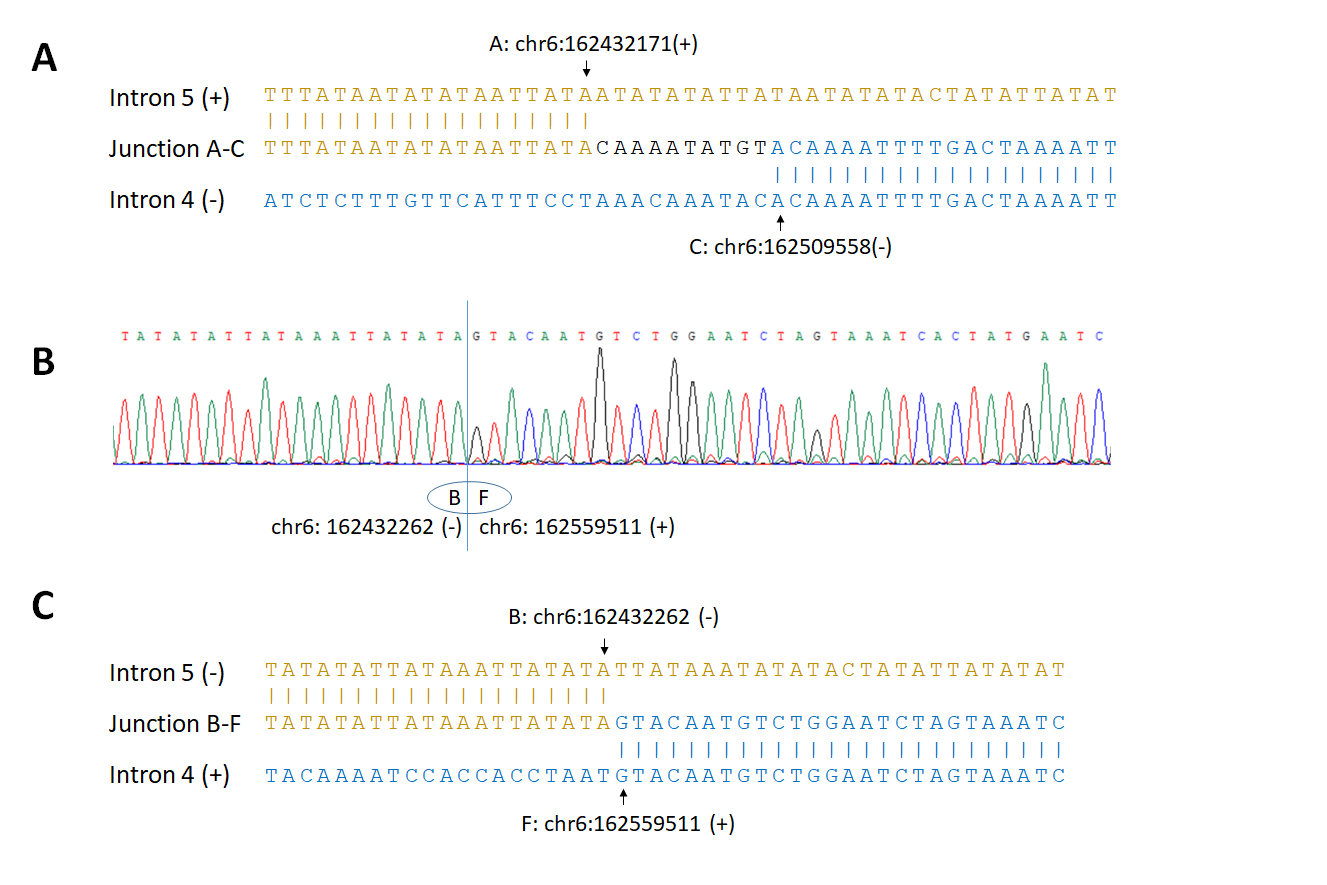
**

**Supplementary Figure 2: The included sequence from intron 5 is flanked by consensus sequences.** The intronic inclusion (93bp) from within the inverted intron 5 is flanked by consensus splice donor and splice acceptor sites (green boxes).


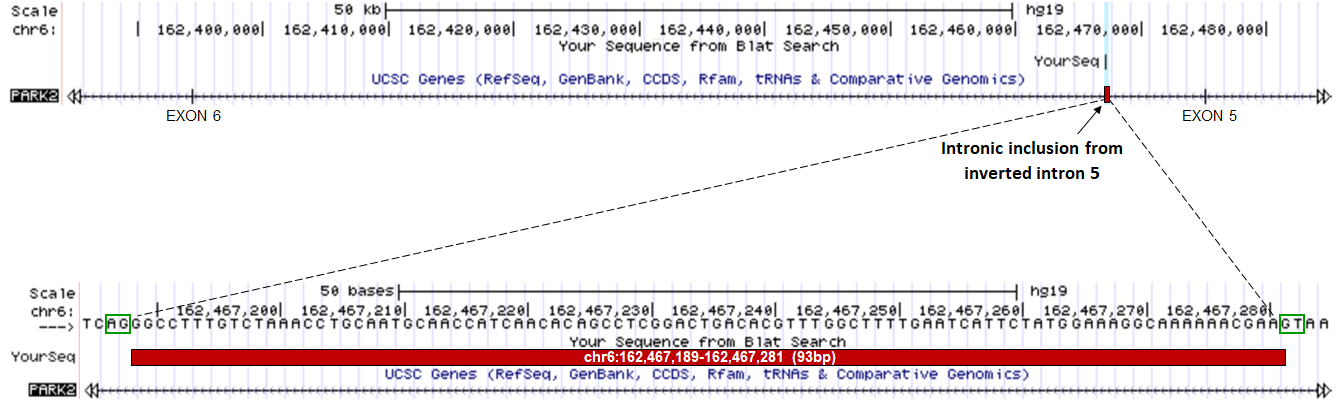

Supplement: fcab197_Supplementary_Data [file fcab197_supplementary_data.docx]
